# Supplementary material for: The catalytic subunit of Plasmodium falciparum casein kinase 2 is essential for gametocytogenesis
Source: Commun Biol. 2021 Mar 12;4:336. doi: 10.1038/s42003-021-01873-0 (PMC7954856; doi:10.1038/s42003-021-01873-0)
Supplement: Supplementary file 2 — Supplementary Information [file 42003_2021_1873_MOESM2_ESM.pdf]

Supplementary Information

The catalytic subunit of *Plasmodium falciparum* casein kinase 2 is essential for gametocytogenesis

Eva Hitz[1,2], Olivia Grüninger[1,2], Armin Passecker[1,2], Matthias Wyss[1,2], Christian Scheurer[1,2], Sergio Wittlin[1,2], Hans-Peter Beck[1,2], Nicolas M. B. Brancucci[1,2], Till S. Voss[1,2,*]

[1]Department of Medical Parasitology and Infection Biology, Swiss Tropical and Public Health Institute, 4051 Basel, Switzerland.

[2]University of Basel, 4001 Basel, Switzerland.

[*]Corresponding author: till.voss@swisstph.ch.

This Supplementary Information file includes:

- Supplementary Figures 1-9
- Supplementary Tables 1 and 2

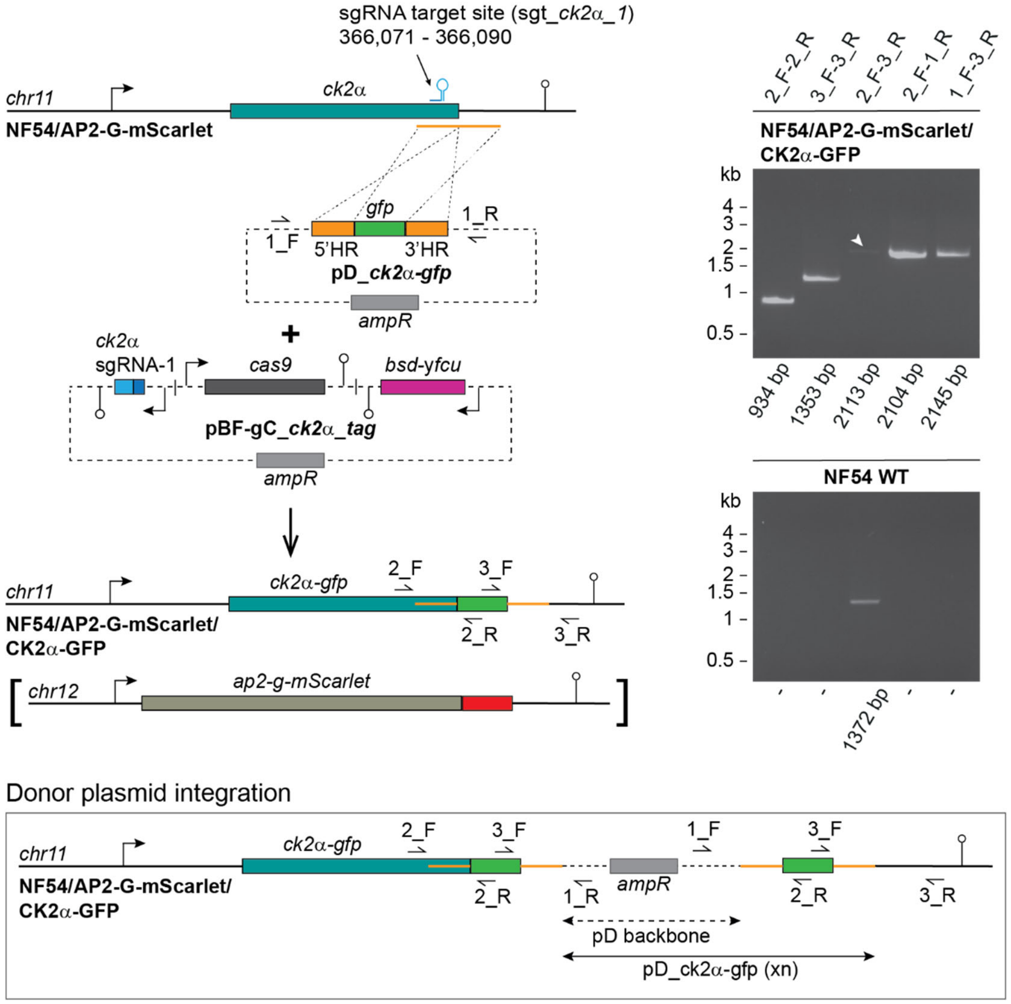

Supplementary Fig 1 CRISPR/Cas9-based engineering of the NF54/AP2-G-mScarlet/CK2α-GFP parasite line. Left: Schematic maps of the WT *pfck2α* locus, the CRISPR/Cas9 suicide and donor plasmids (pBF-gC_*ck2α_tag* and pD_*ck2α-gfp*) used to generate the NF54/AP2-G-mScarlet/CK2α-GFP parasite line and the modified *pfck2α* locus after editing (the modified *pfap2-g-mScarlet* locus in NF54/AP2-G-mScarlet parasites (Brancucci et al., manuscript in preparation) is schematically depicted in brackets) (bottom). A schematic illustrating a donor plasmid concatamer integration event by double-crossover recombination is shown in the box below (for simplicity integration of a tandem assembly is shown). Names and relative binding sites of the primers used for diagnostic PCRs are indicated. Right: PCRs performed on gDNA of NF54/AP2-G-mScarlet/CK2α-GFP parasites confirm correct tagging of the *pfck2α* gene (PCR reactions: 2_F-2_R, 3_F-3_R, 2_F-3_R) and donor plasmid concatamer integration in a subset of parasites in the population (PCR reactions: 3_F-1_R and 1_F-4_R). PCRs performed on gDNA of NF54 WT parasites serve as control.

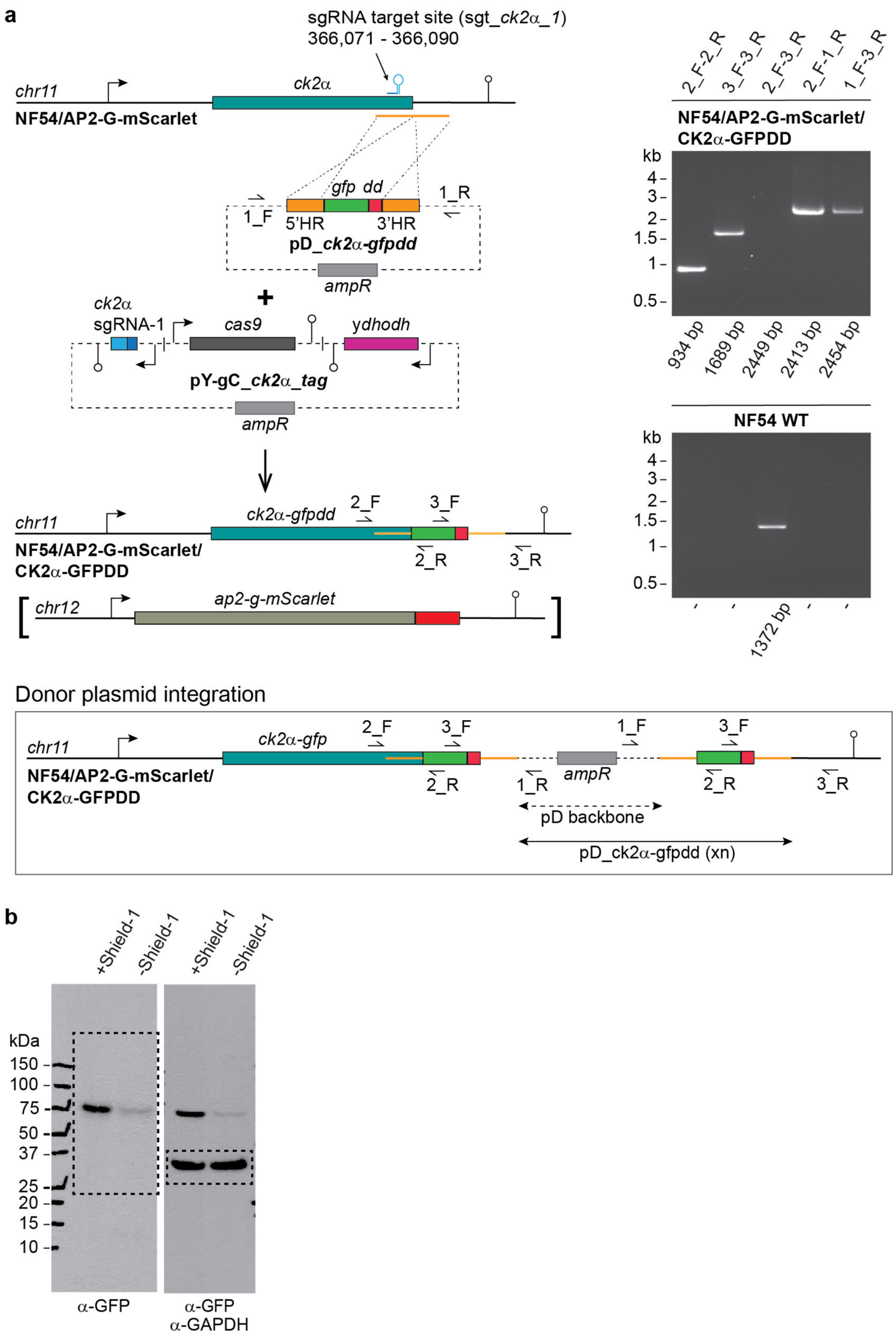

Supplementary Fig 2 CRISPR/Cas9-based engineering of the NF54/AP2-G-mScarlet/CK2α-GFPDD parasite line and full-size Western blot of the blot section shown in Fig. 2. a Left: Schematic maps of the WT *pfck2α* locus, the CRISPR/Cas9 suicide and donor plasmids (pY-gC_*ck2α_tag* and

pD_*ck2α-gfpdd*) used to generate the NF54/AP2-G-mScarlet/CK2α-GFPDD parasite line and the modified *pfck2α* locus after editing (the modified *pfap2-g-mScarlet* locus in NF54/AP2-G-mScarlet parasites (Brancucci et al., manuscript in preparation) is schematically depicted in brackets) (bottom). A schematic illustrating a donor plasmid concatamer integration event by double-crossover recombination is shown in the box below (for simplicity integration of a tandem assembly is shown). Names and relative binding sites of the primers used for diagnostic PCRs are indicated. Right: PCRs performed on gDNA of NF54/AP2-G-mScarlet/CK2α-GFPDD parasites confirm correct tagging of the *pfck2α* gene (PCR reactions: 2_F-2_R, 3_F-3_R). The PCRs also detected donor plasmid concatamer integration in all parasites in the population (PCR reactions: 2_F-1_R and 1_F-3_R), explained by the lack of a PCR product spanning the full modified locus (PCR reaction: 2_F-3_R). PCRs performed on gDNA of NF54 WT parasites serve as control. **b** Full-size Western blot showing expression of PfCK2α-GFPDD in NF54/AP2-G-mScarlet/CK2α-GFPDD schizonts cultured in presence (+Shield-1) and absence (-Shield-1) of Shield-1. Parasites were split (±Shield-1) 40 hours before sample collection. Protein lysates derived from an equal number of parasites were loaded per lane. The membrane was first probed with α-GFP antibodies (left) and subsequently with α-GAPDH control antibodies (right). MW PfCK2α-GFPDD = 78.7 kDa, MW loading control PfGAPDH = 36.6 kDa.

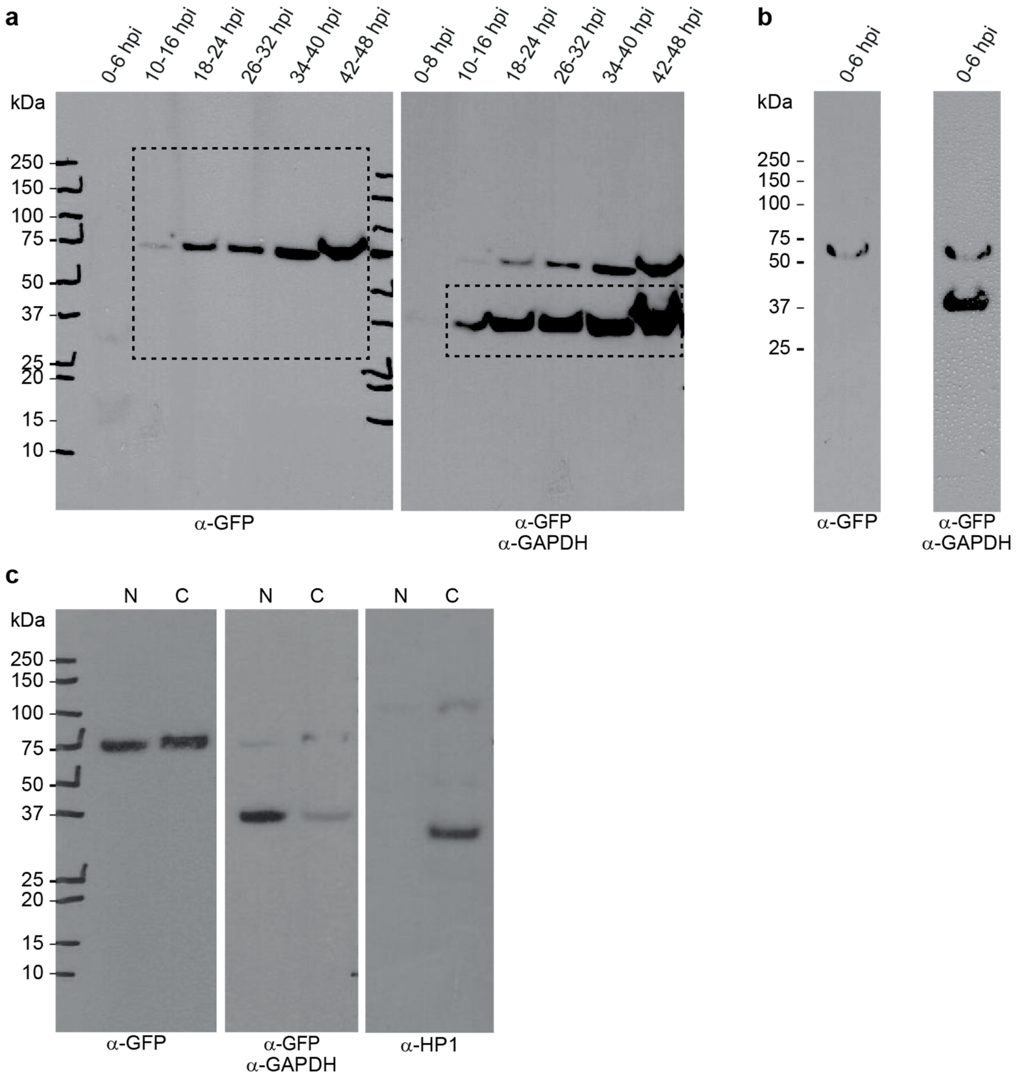

Supplementary Fig 3 Full-size Western blots of the blot sections shown in Fig. 1 and of cytoplasmic and nuclear protein fractions. a Full-size Western blot showing expression of PfCK2α-GFP in NF54/AP2-G-mScarlet/CK2α-GFP parasites at several time points during the IDC. Protein lysates derived from an equal number of parasites were loaded per lane. The membrane was first probed with α-GFP antibodies (left) and subsequently with α-GAPDH control antibodies (right). **b** Full-size Western blot showing expression of PfCK2α-GFP in early ring stage parasites (0-6 hpi) (three-fold higher amount of lysate loaded compared to panel a). The membrane was first probed with α-GFP antibodies (left) and subsequently with α-GAPDH control antibodies (right). **c** Full-size Western blot showing PfCK2α-GFP expression in nuclear and cytoplasmic extracts of NF54/AP2-G-mScarlet/CK2α-GFP parasites. The membrane was first probed with α-GFP antibodies (left) and subsequently with α-GAPDH control

antibodies (center). After stripping off the α-GFP/α-GAPDH and secondary antibodies, the membrane was re-probed using α-HP1 control antibodies. Nuclear and cytoplasmic protein extracts derived from the same parasite sample and equal proportions of both extracts were loaded. C, cytoplasmic extract; N, nuclear extract. MW PfCK2α-GFP = 66.8 kDa, MW loading control PfGAPDH = 36.6 kDa, MW PfHP1 = 31 kDa.

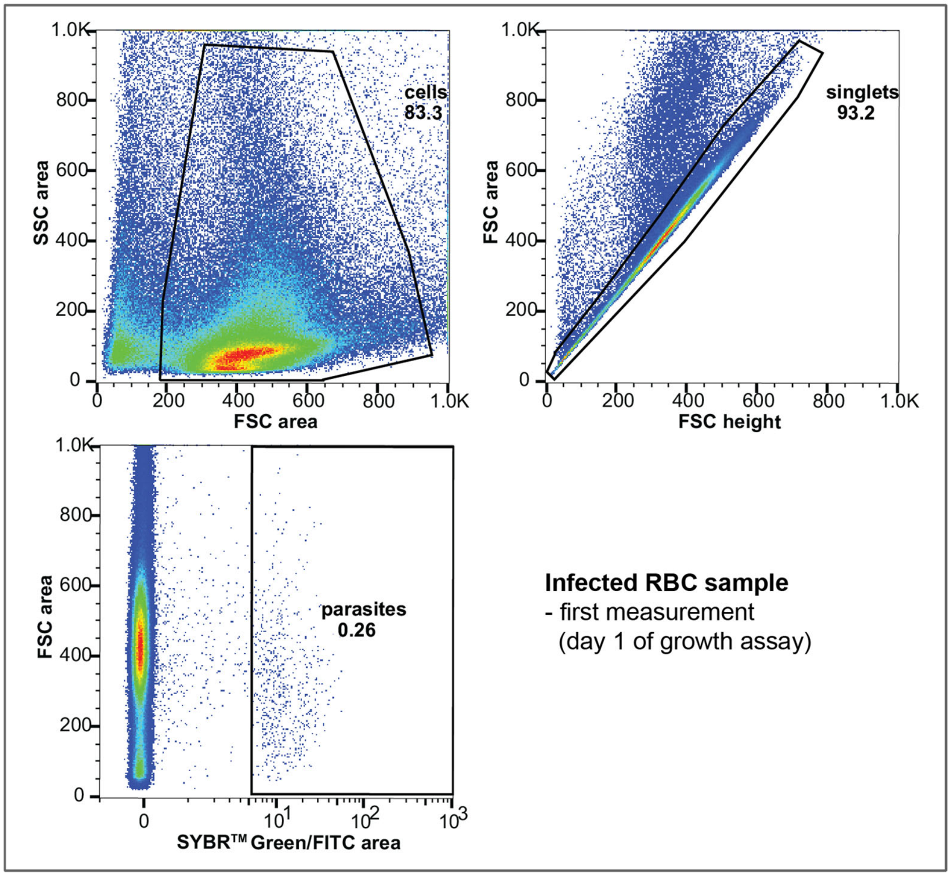
cells
83.3
SSC area
FSC area
singlets
93.2
FSC area
FSC height
parasites
0.26
FSC area
SYBR™ Green/FITC area
Infected RBC sample
- first measurement
(day 1 of growth assay)

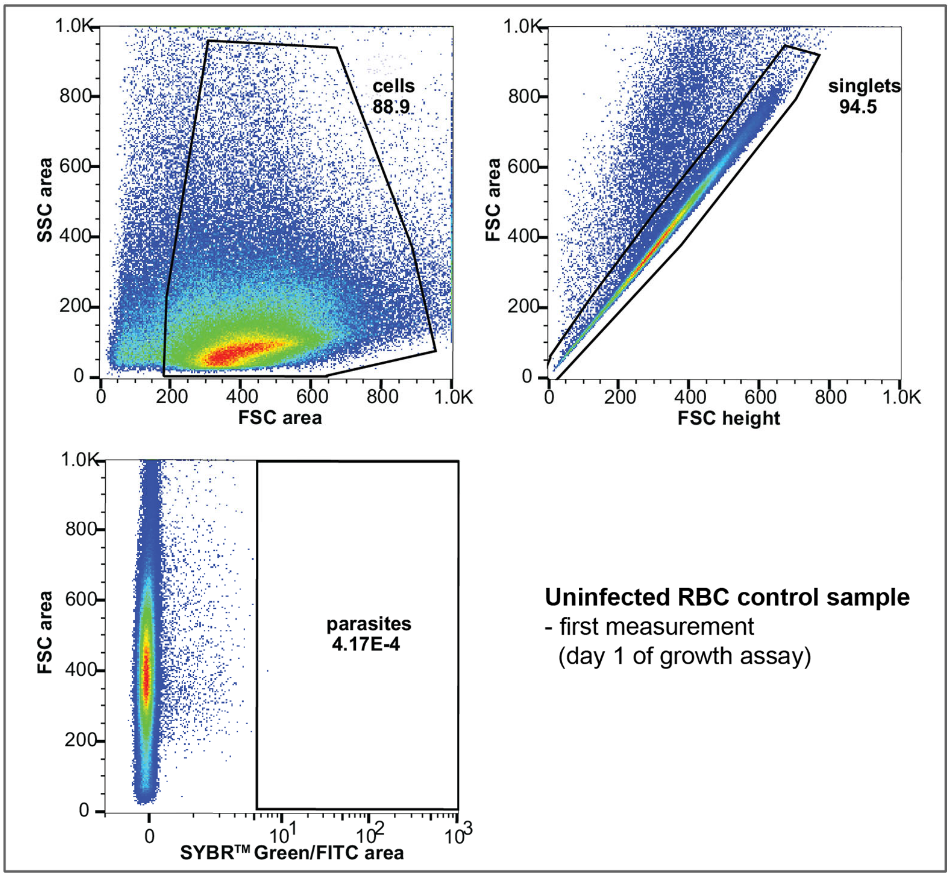
cells
88.9
SSC area
FSC area
singlets
94.5
FSC area
FSC height
parasites
4.17E-4
FSC area
SYBR™ Green/FITC area
Uninfected RBC control sample
- first measurement
(day 1 of growth assay)

Supplementary Fig 4 Gating strategy of flow cytometry data obtained from parasite multiplication assays. Representative flow cytometry plots of an infected (top frame; NF54/AP2-G-mScarlet/CK2α-GFPDD +Shield-1) and an uninfected RBC control sample (bottom frame) on the first day of the multiplication assay are shown. The first plot (top left) for both samples shows the gate set to remove small debris (smaller than cell size), keeping the "cells" used for further gating. The "cells" population was gated to remove doublets (single measurement events consisting of two cells), keeping "singlets" used for further gating (top right). Finally, SYBR green fluorescence intensity was used to separate uninfected from infected RBCs (bottom left). The numbers indicate the percentage of cells included in the infected RBC or "parasites" gate, directly reflecting the parasitaemia of the sample. This gating strategy was applied to the flow cytometry data shown in Figs. 2b and 4c.

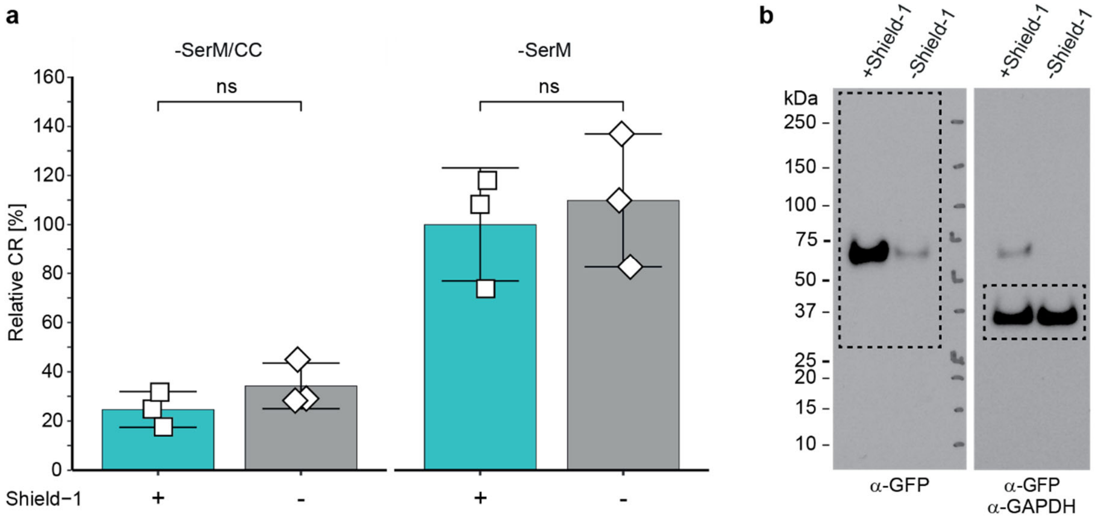

Supplementary Fig 5 Sexual commitment rates of NF54/AP2-G-mScarlet/CK2α-GFPDD parasites and full-size Western blot of the blot section shown in Fig. 3b. a Relative sexual commitment rates of parasites cultured in serum-free medium either in presence (–SerM/CC) or absence (–SerM) of 2 mM choline chloride [1]. Parasites cultured in presence (+Shield-1; turquoise) or absence (-Shield-1; grey) of Shield-1 are compared. Parasites were split (±Shield-1) at 0-6 hpi, transferred to –SerM or –SerM/CC medium conditions at 18-24 hpi and sexual commitment rates were assessed in the progeny at 18-24 hpi by quantifying AP2-G-mScarlet-positivity among all iRBCs (Hoechst-positive cells) by high content imaging (>3,000 iRBCs counted per experiment). The mean ±SD of three biological replicates is shown. Data points for individual replicates are represented by open squares. ns, not significant (paired two-tailed Student's t test). CR, commitment rate. **b** Full-size Western blot showing expression of PfCK2α-GFPDD in NF54/AP2-G-mScarlet/CK2α-GFPDD stage V gametocytes cultured in presence (+Shield-1) and absence (-Shield-1) of Shield-1. Parasites were split (±Shield-1) at 0-6 hpi, transferred to –SerM at 18-24 hpi to induce sexual commitment and subsequently cultured in presence or absence of Shield-1 until stage V gametocytes were obtained. From the ring stage progeny (asexual/sexual ring stages) until day six of gametocytogenesis, parasites were cultured in presence of 50 mM GlcNAc to eliminate asexual parasites. Protein lysates derived from an equal number of parasites were loaded per lane. The membrane was first probed with α-GFP antibodies (left) and subsequently with α-GAPDH control antibodies (right). MW PfCK2α-GFPDD = 78.7 kDa, MW loading control PfGAPDH = 36.6 kDa.

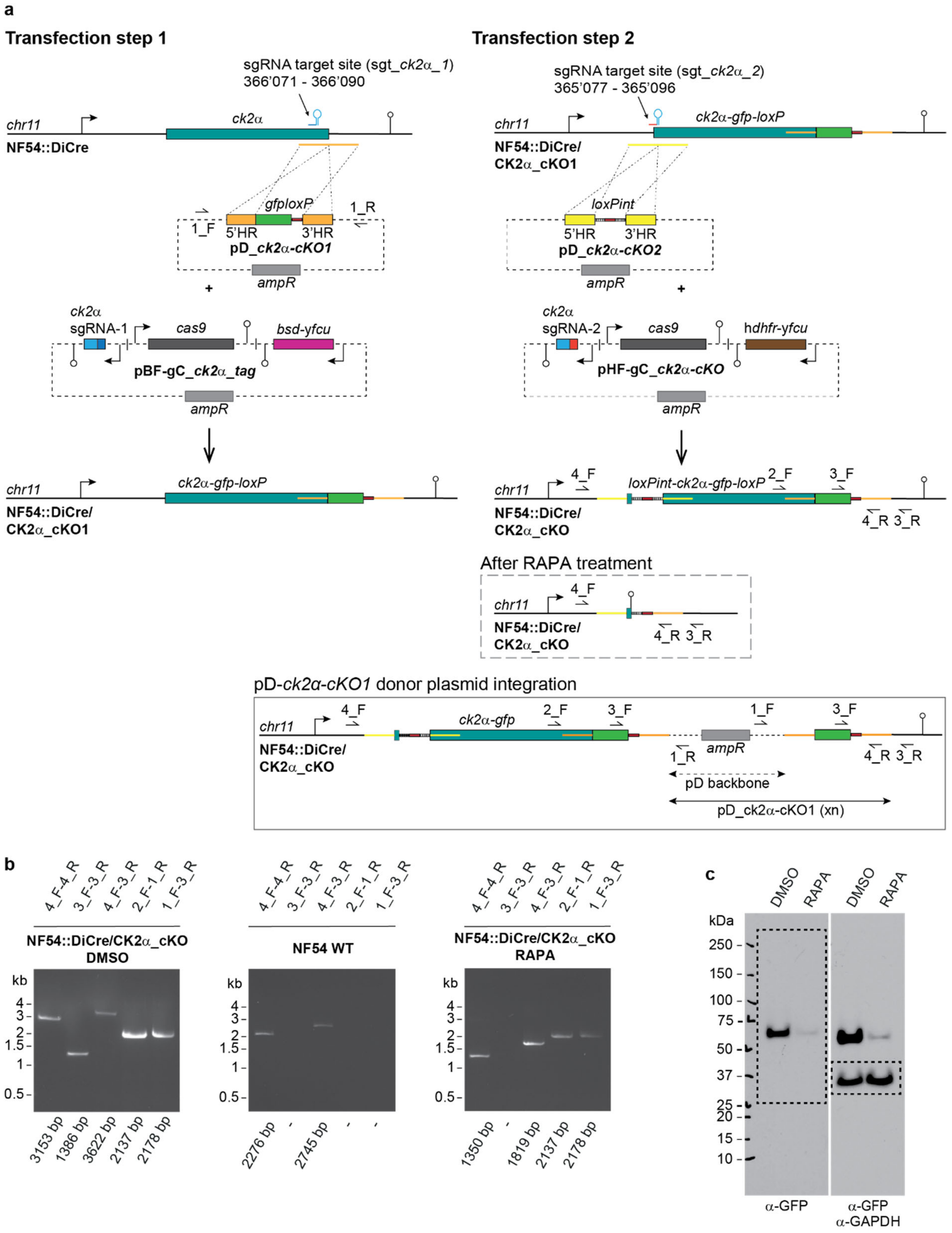

Supplementary Fig 6 CRISPR/Cas9-based engineering of the NF54::DiCre/CK2α_cKO parasite line. a Left: Schematic maps of the WT *pfck2α* locus, the CRISPR/Cas9 suicide and donor plasmids (pBF-gC_*ck2α_tag* and pD_*ck2α-cKO1*) used for the transfection of NF54::DiCre parasites[2] and the

modified *pfck2α* locus after editing. Right: Schematic maps the *pfck2a-gfp-loxP* locus, the CRISPR/Cas9 suicide and donor plasmids (pHF-gC_*ck2α-cKO* and pD_*ck2α-cKO2*) used for the transfection of NF54::DiCre/CK2α_cKO1 parasites and the modified *pfck2α* locus after editing, resulting in the generation of the NF54::DiCre/CK2α_cKO parasite line. The schematic map in the dashed box below shows the *pfck2α* locus after DiCre-mediated excision of the *pfck2α-gfp* gene. The schematic map in the box at the bottom illustrates a pD_*ck2α-cKO1* donor plasmid concatamer integration event by double-crossover recombination (for simplicity integration of a tandem assembly is shown). Names and relative binding sites of the primers used for diagnostic PCRs are indicated. **b** Left: PCRs performed on gDNA of NF54::DiCre/CK2α_cKO parasites treated with DMSO confirm correct editing of the locus (PCR reactions: 4_F-4_R, 3_F-3_R, 4_F-3_R) and reveal integration of pD_*ck2α-cKO1* donor plasmid concatamers in a subset of parasites in the population (PCR reactions: 2_F-1_R and 1_F-3_R). Center: Control PCRs performed on gDNA of NF54 WT parasites. Right: PCRs performed on gDNA of NF54::DiCre/CK2α_cKO parasites treated with RAPA at 0-6 hpi for four hours and harvested 40 hours later (40-46 hpi) confirm successful excision of the *pfck2α-gfp* gene upon action of the DiCre recombinase (PCR reactions: 4_F-4_R, 3_F-3_R, 4_F-3_R). Donor plasmid concatamer integration events were still detectable suggesting that DiCre-mediated excision was not 100% efficient (PCR reactions: 2_F-1_R and 1_F-3_R). However, these parasites were of very low abundance since even seven days after RAPA treatment no viable parasites were observed (Fig. 4). RAPA, rapamycin. **c** Full-size Western blot of the blot sections shown in Fig. 4. Full-size Western blot showing expression of PfCK2α-GFP in PfCK2α-GFP KO (RAPA) and control (DMSO) schizonts. Parasites were split and exposed for four hours to either RAPA or DMSO 40 hours before sample collection. Protein lysates derived from an equal number of parasites were loaded per lane. The membrane was first probed with α-GFP antibodies (left) and subsequently with α-GAPDH control antibodies (right). MW PfCK2α-GFP = 66.8 kDa, MW loading control PfGAPDH = 36.6 kDa. RAPA, rapamycin.

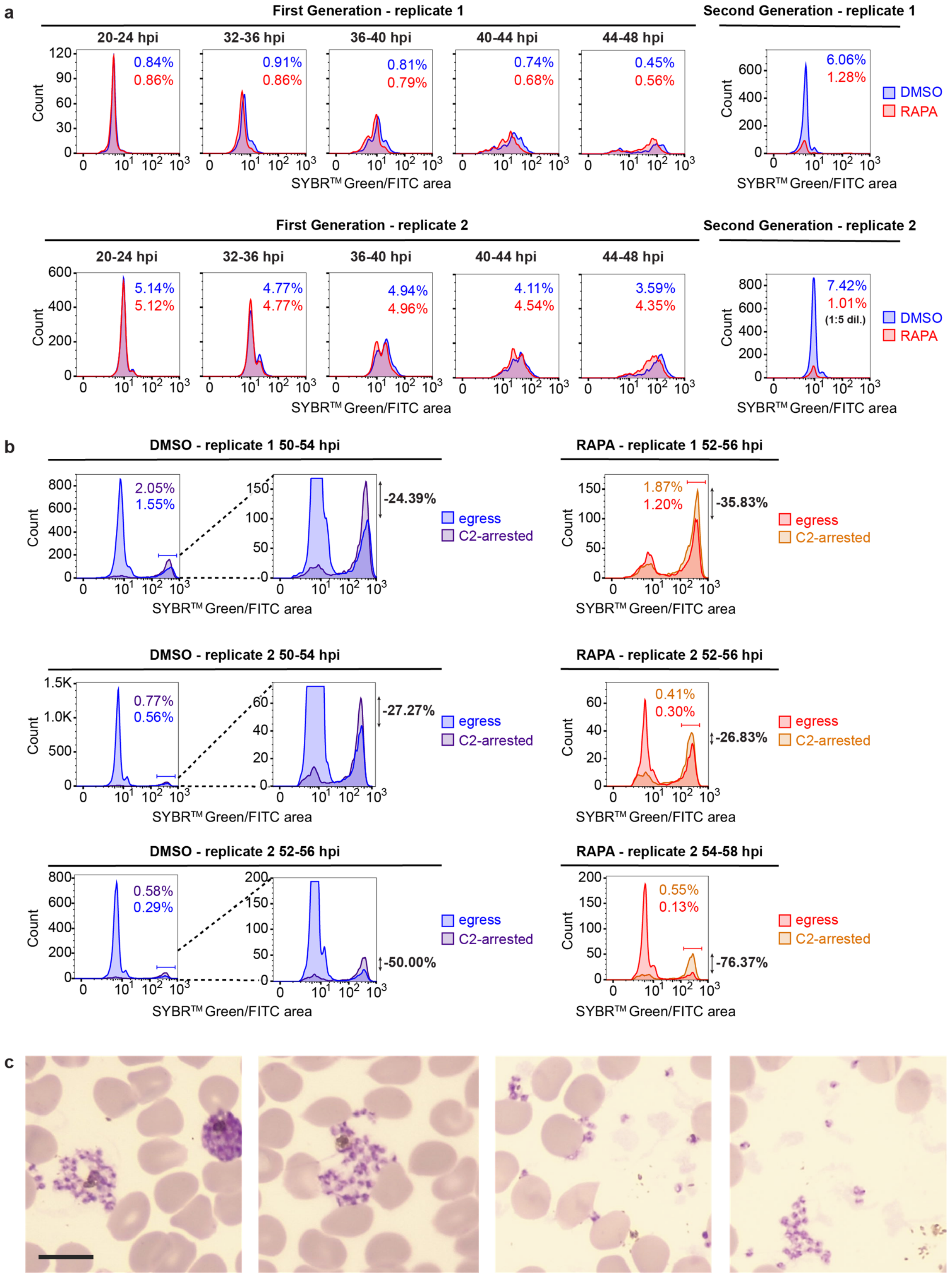

Supplementary Fig 7 Parasite progression through schizogony and merozoite egress and invasion in NF54::DiCre/CK2α-GFP KO (RAPA) and control (DMSO) parasites. a Flow cytometry time course experiment showing parasite progression through schizogony as determined by quantifying the

increase in genome content based on SYBR Green intensity (x-axis). Parasites were split at 0-4 hpi and treated for four hours with either RAPA (red) or DMSO (blue) before sampling five consecutive time points from 20-24 hpi (late ring stages) to 44-48 hpi (mature schizonts) in generation 1 and one ring stage time point in the subsequent progeny (generation 2). Numbers indicate total parasitaemia. Two independent time course experiments are shown. Note that for replicate 2 the parasitaemia has been diluted 1:5 prior to schizont rupture (1:5 dil.). hpi, hours post-invasion. **b** Flow cytometry experiment showing merozoite egress as determined by quantifying the percentage of schizont-infected RBCs based on SYBR reen intensity. Parasites were split at 0-4 hpi, treated for four hours with either RAPA or DMSO and exposed to C2 from 36-40 hpi onwards. Sampling was performed at 50-54 to 54-58 hpi from C2-arrested populations (RAPA, orange; DMSO, purple) and 45-90 min after release from C2-mediated developmental arrest (RAPA, red; DMSO, blue). Numbers indicate total schizontaemia. The relative reduction in schizontaemia after release from C2-mediated arrest compared to the C2-arrested control is shown to the right of the graphs. Two independent experiments are shown. hpi, hours post-invasion. **c** Representative images from Giemsa-stained thin blood smears prepared from RAPA-treated mature NF54::DiCre/CK2α-GFP KO schizonts 20 min after reversal of C2-mediated developmental arrest, showing ruptured schizonts and released merozoites. Scale bar = 10 µm.

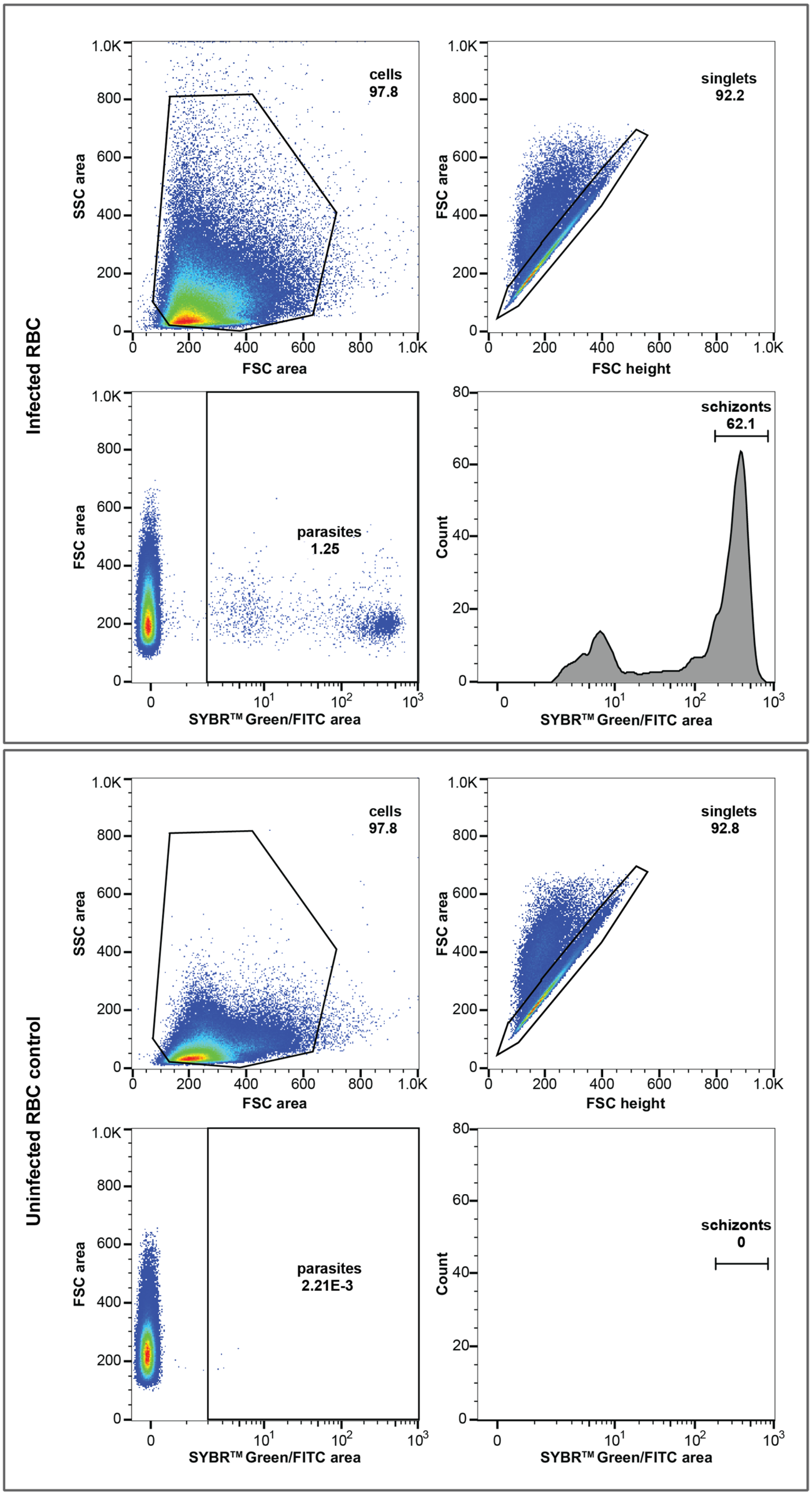
Infected RBC
cells
97.8
SSC area
FSC area
singlets
92.2
FSC area
FSC height
parasites
1.25
FSC area
SYBR™ Green/FITC area
schizonts
62.1
Count
SYBR™ Green/FITC area
Uninfected RBC control
cells
97.8
SSC area
FSC area
singlets
92.8
FSC area
FSC height
parasites
2.21E-3
FSC area
SYBR™ Green/FITC area
schizonts
0
Count
SYBR™ Green/FITC area

Supplementary Fig 8 Gating strategy of flow cytometry data obtained from parasite schizogony and merozoite egress assays. Representative flow cytometry plots of an infected (top frame; NF54::DiCre/CK2α_cKO, DMSO-treated control) and an uninfected RBC control sample (bottom frame). The first plot (top left) for both samples shows the gate set to remove small debris (smaller than cell size), keeping the “cells” used for further gating. The “cells” population was gated to remove doublets (single measurement events consisting of two cells), keeping “singlets” used for further gating (top right). SYBR green fluorescence intensity was used to separate uninfected from infected RBCs ("parasites"; bottom left) or mature schizonts from other iRBCs ("schizonts"; botttom right). The numbers indicate the percentage of cells included in the “parasites” or "schizonts" gates, directly reflecting the parasitaemia or schizontaemia of the sample, respectively. This gating strategy was applied to the flow cytometry data shown in Supplementary Fig. 7.

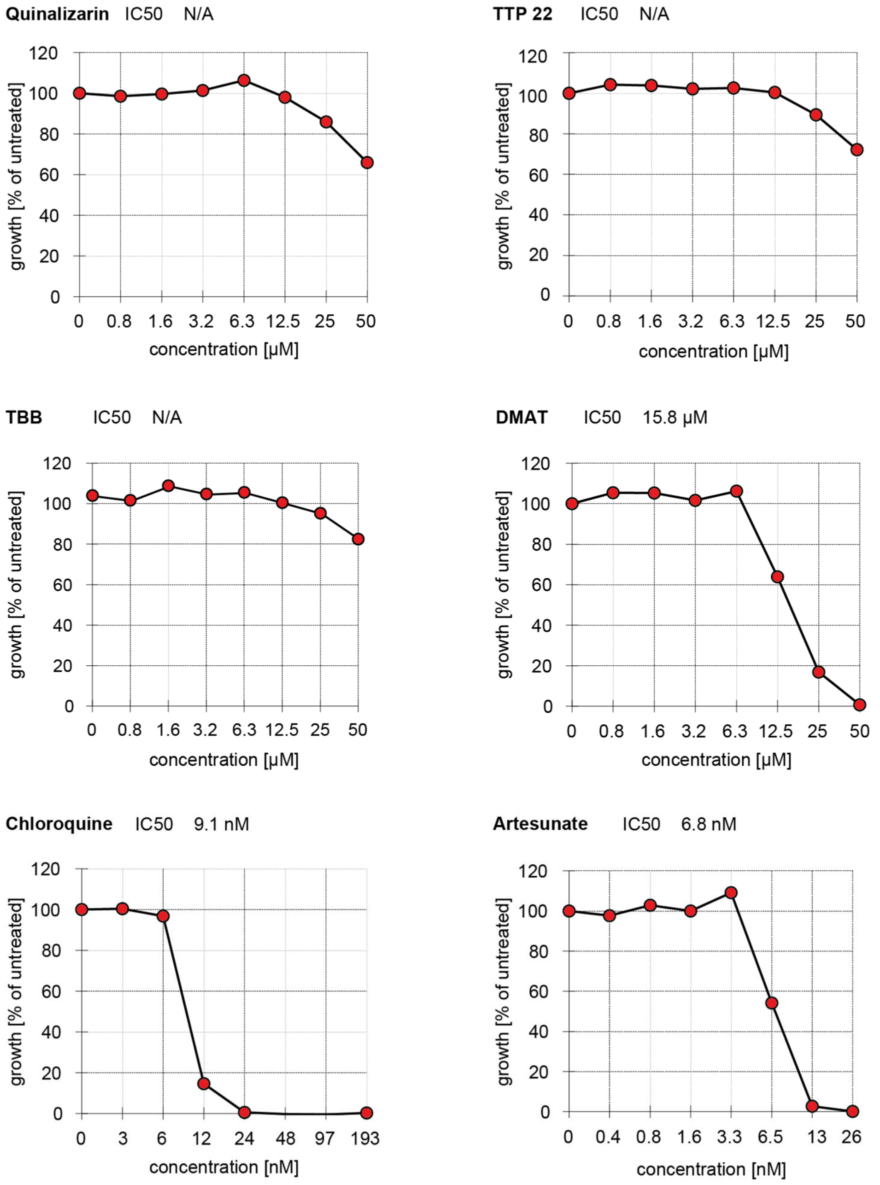

Supplementary Fig 9 Growth inhibition curves of compounds tested on NF54 asexual parasites in a $[^3H]$ hypoxanthine incorporation assay. Growth inhibition curves of the four human CK2α inhibitors Quinalizarin, TTP 22, TBB and DMAT. Maximal concentration of compounds tested: 50 µM. Growth inhibition curves of the two antimalarial control compounds Chloroquine and Artesunate are shown at the bottom. Maximal concentrations for Chloroquine and Artesunate were 193 nM and 26 nM, respectively. The means of two biological replicates are shown. One of the biological replicate experiments was performed in two technical replicates. The fifty percent inhibitory concentration (IC_{50}) is indicated above each graph. N/A, not applicable.

Supplementary Table 1 Oligonucleotides used for cloning of CRISPR/Cas9 transfection constructs. Names of oligonucleotides, plasmids and cell lines as well as oligonucleotide sequences are shown. Sequences essential for Gibson assembly reactions (Gibson overhangs) or for T4 DNA ligase-dependent cloning of double-stranded sgRNA-encoding fragments (5' and 3' overhangs) are highlighted with capital or italicized capital letters, respectively.

**Oligo-nucleotide**	**Sequence 5' → 3'**	**Plasmids**	**Cell line**
Y_F	GACGGAGCTCGAATTCGcgaagatctgtcttaagcattttg	pY-gC mother plasmid	-
Y_R	CTTTTCTTCTTCAGGGTAGCccatggagttatcgatatgaattc	pY-gC mother plasmid	-
B_F	CTCGTTTAAGACCTGAAATCaaagttatcg	pY-gC mother plasmid	-
B_R	GATTTCAGGTCTTAAACGAGtgtaaaatgcacg	pY-gC mother plasmid	-
sgt_ck2a_1_F	*TATT*tcacggacttctctaaagta	pBF-gC_ck2α_tag; pY-gC_ck2α_tag	NF54/AP2-G-mScarlet/CK2α-GFP; NF54/AP2-G-mScarlet/CK2α-GFPDD; NF54::DiCre/CK2α_cKO
sgt_ck2a_1_R	*AAAC*tactttagagaagtccgtga	pBF-gC_ck2α_tag; pY-gC_ck2α_tag	NF54/AP2-G-mScarlet/CK2α-GFP; NF54/AP2-G-mScarlet/CK2α-GFPDD; NF54::DiCre/CK2α_cKO
sgt_ck2a_2_F	*TATT*aaaagagacaggaataatgt	pHF-gC_ck2α-cKO	NF54::DiCre/CK2α_cKO
sgt_ck2a_2_R	*AAAC*acattattcctgtctctttt	pHF-gC_ck2α-cKO	NF54::DiCre/CK2α_cKO
PCRA_F	CTGGCGTAATAGCGAAGAGG	pD_ck2α-gfp; pD_ck2α-gfpdd; pD_ck2α-cKO1; pD_ck2α-cKO2	NF54/AP2-G-mScarlet/CK2α-GFP; NF54/AP2-G-mScarlet/CK2α-GFPDD; NF54::DiCre/CK2α_cKO
PCRA_R	CATTAATGAATCGGCCAACG	pD_ck2α-gfp; pD_ck2α-gfpdd; pD_ck2α-cKO1; pD_ck2α-cKO2	NF54/AP2-G-mScarlet/CK2α-GFP; NF54/AP2-G-mScarlet/CK2α-GFPDD; NF54::DiCre/CK2α_cKO
H1_F	CGTTGGCCGATTCATTAATGctggtcaagaatataatgttcgtg	pD_ck2α-gfp; pD_ck2α-gfpdd; pD_ck2α-cKO1	NF54/AP2-G-mScarlet/CK2α-GFP; NF54/AP2-G-mScarlet/CK2α-GFPDD; NF54::DiCre/CK2α_cKO
H1_R	CTCTCTTCTCTAACCTCGCGgaaatagggatgctccatggcttcc	pD_ck2α-gfp; pD_ck2α-gfpdd	NF54/AP2-G-mScarlet/CK2α-GFP NF54/AP2-G-mScarlet/CK2α-GFPDD
G_F	CGCGAGGTTAGAGAAGAGAGtggatccagtggaatgagtaaag	pD_ck2α-gfp; pD_ck2α-gfpdd	NF54/AP2-G-mScarlet/CK2α-GFP NF54/AP2-G-mScarlet/CK2α-GFPDD
G_R	GATTCCTCACGTTATTTGTATAGttcatccatgccatg	pD_ck2α-gfp	NF54/AP2-G-mScarlet/CK2α-GFP
GD_R	GATTCCTCACGTCATTCTAAttttagaagctccacacgg	pD_ck2α-gfpdd	NF54/AP2-G-mScarlet/CK2α-GFPDD
H2_F	CTATACAAATAACGTGAGGAATCataataataataaaaaaaaatg	pD_ck2α-gfp	NF54/AP2-G-mScarlet/CK2α-GFP
H2D_F	TTAGAATGACGTGAGGAATCataataataataaaaaaaaatg	pD_ck2α-gfpdd	NF54/AP2-G-mScarlet/CK2α-GFPDD
H2_R	CCTCTTCGCTATTACGCCAGttgagcacatttttctctataagg	pD_ck2α-gfp; pD_ck2α-gfpdd; pD_ck2α-cKO1	NF54/AP2-G-mScarlet/CK2α-GFP; NF54/AP2-G-mScarlet/CK2α-GFPDD; NF54::DiCre/CK2α_cKO
loxP1_R	CGTATAATGTATGCTATACGaagttatttatttgtatagttcatccatgcc	pD_ck2α-cKO1	NF54::DiCre/CK2α_cKO
loxP1_F	CGTATAGCATACATTATACGaagttatgtgaggaatcataataataataaaaaaaaatg	pD_ck2α-cKO1	NF54::DiCre/CK2α_cKO
H3_F	CGTTGGCCGATTCATTAATGgataacaaaaatataaatcaagatcgt	pD_ck2α-cKO2	NF54::DiCre/CK2α_cKO
H3_R	TTTTTTATTTACCTAACAGACATTATTcctgtctctttttaaaaacg	pD_ck2α-cKO2	NF54::DiCre/CK2α_cKO
loxPINT_F	AATAATGTCTGTTAGGTAAATAAAAAAaataatatacaataacttcgtatag	pD_ck2α-cKO2	NF54::DiCre/CK2α_cKO
loxPINT_R	GGTATATAAATTTTTTTATTAATTGAGCTAAAAGAATATAAAATATATAAATATatatatatatac	pD_ck2α-cKO2	NF54::DiCre/CK2α_cKO
H4_F	ATATTTATATATTTTATATTCTTTTAGCTCAATTAATAAAAAAATTTATATACCaaaattttatg	pD_ck2α-cKO2	NF54::DiCre/CK2α_cKO
H4_R	CTTCGCTATTACGCCAGcagctagaccccaatcaa	pD_ck2α-cKO2	NF54::DiCre/CK2α_cKO

Supplementary Table 2 Primers used for PCRs on gDNA of transgenic parasite lines. Primer names, sequences and names of the transgenic cell lines are shown.

Primer	Sequence 5' → 3'	Cell line
1_F	gcgaggaagcggaagagc	NF54/AP2-G-mScarlet/CK2α-GFP; NF54/AP2-G-mScarlet/CK2α-GFPDD; NF54::DiCre/CK2α_cKO
1_R	attcgccattcaggctgc	NF54/AP2-G-mScarlet/CK2α-GFP; NF54/AP2-G-mScarlet/CK2α-GFPDD; NF54::DiCre/CK2α_cKO
2_F	attaattgattggggtctagc	NF54/AP2-G-mScarlet/CK2α-GFP; NF54/AP2-G-mScarlet/CK2α-GFPDD; NF54::DiCre/CK2α_cKO
2_R	gtgtgagttatagttgtattcc	NF54/AP2-G-mScarlet/CK2α-GFP; NF54/AP2-G-mScarlet/CK2α-GFPDD
3_F	ggttatgtacaggaaagaac	NF54/AP2-G-mScarlet/CK2α-GFP; NF54/AP2-G-mScarlet/CK2α-GFPDD; NF54::DiCre/CK2α_cKO
3_R	gaaataagaaataaaaaataagaaataagaaaac	NF54/AP2-G-mScarlet/CK2α-GFP; NF54/AP2-G-mScarlet/CK2α-GFPDD; NF54::DiCre/CK2α_cKO
4_F	gtcataccacatttccaatc	NF54::DiCre/CK2α_cKO
4_R	cgtgcataaatcatacacac	NF54::DiCre/CK2α_cKO

Supplementary References

1 Brancucci, N. M. B. *et al.* Lysophosphatidylcholine Regulates Sexual Stage Differentiation in the Human Malaria Parasite Plasmodium falciparum. *Cell* **171**, 1532-1544 (2017).

2 Tiburcio, M. *et al.* A Novel Tool for the Generation of Conditional Knockouts To Study Gene Function across the Plasmodium falciparum Life Cycle. *MBio* **10**, e01170-19 (2019).
