# Supplementary material for: The catalytic subunit of Plasmodium falciparum casein kinase 2 is essential for gametocytogenesis
Source: Commun Biol. 2021 Mar 12;4:336. doi: 10.1038/s42003-021-01873-0 (PMC7954856; doi:10.1038/s42003-021-01873-0)
Supplement: Supplementary file 5 — Reporting Summary [file 42003_2021_1873_MOESM5_ESM.pdf]

nature research

Corresponding author(s): Till Voss
Last updated by author(s): 11.02.2021

Reporting Summary

Nature Research wishes to improve the reproducibility of the work that we publish. This form provides structure for consistency and transparency in reporting. For further information on Nature Research policies, see our Editorial Policies and the Editorial Policy Checklist.

Statistics

For all statistical analyses, confirm that the following items are present in the figure legend, table legend, main text, or Methods section.

n/a	Confirmed	
☐	☒	The exact sample size (*n*) for each experimental group/condition, given as a discrete number and unit of measurement
☐	☒	A statement on whether measurements were taken from distinct samples or whether the same sample was measured repeatedly
☐	☒	The statistical test(s) used AND whether they are one- or two-sided<br>*Only common tests should be described solely by name; describe more complex techniques in the Methods section.*
☒	☐	A description of all covariates tested
☒	☐	A description of any assumptions or corrections, such as tests of normality and adjustment for multiple comparisons
☐	☒	A full description of the statistical parameters including central tendency (e.g. means) or other basic estimates (e.g. regression coefficient) AND variation (e.g. standard deviation) or associated estimates of uncertainty (e.g. confidence intervals)
☐	☒	For null hypothesis testing, the test statistic (e.g. *F*, *t*, *r*) with confidence intervals, effect sizes, degrees of freedom and *P* value noted<br>*Give P values as exact values whenever suitable.*
☒	☐	For Bayesian analysis, information on the choice of priors and Markov chain Monte Carlo settings
☒	☐	For hierarchical and complex designs, identification of the appropriate level for tests and full reporting of outcomes
☒	☐	Estimates of effect sizes (e.g. Cohen's *d*, Pearson's *r*), indicating how they were calculated

Our web collection on statistics for biologists contains articles on many of the points above.

Software and code

Policy information about availability of computer code

Data collection	Microscopy images obtained with the Leica DM5000 B fluorescence microscope were acquired with the Leica application suite software (LAS 4.9.0) and processed using Adobe Photoshop CC 2018. Microscopy images obtained with the ImageXpress® Micro XLS microscope (Molecular Devices) were acquired and analysed with the MetaXpress software (version 6.5.4.532, Molecular Devices). Flow cytometry data obtained with the MACS Quant Analyzer 10 (Miltenyi Biotec) were analysed with the FlowJo software (v10.6.1).
Data analysis	Data were analysed and plotted using RStudio Version 1.1.456 and package ggplot2.

For manuscripts utilizing custom algorithms or software that are central to the research but not yet described in published literature, software must be made available to editors and reviewers. We strongly encourage code deposition in a community repository (e.g. GitHub). See the Nature Research guidelines for submitting code & software for further information.

Data

Policy information about availability of data

All manuscripts must include a data availability statement. This statement should provide the following information, where applicable:

- Accession codes, unique identifiers, or web links for publicly available datasets
- A list of figures that have associated raw data
- A description of any restrictions on data availability

The source data for all the graphs and charts in the main figures are present in Supplementary Data 1 and any other remaining information can be available from the corresponding author upon reasonable request.

Field-specific reporting

Please select the one below that is the best fit for your research. If you are not sure, read the appropriate sections before making your selection.

☒ Life sciences ☐ Behavioural & social sciences ☐ Ecological, evolutionary & environmental sciences

For a reference copy of the document with all sections, see nature.com/documents/nr-reporting-summary-flat.pdf

Life sciences study design

All studies must disclose on these points even when the disclosure is negative.

Sample size	Sample-size calculation was not required for this work. Sample sizes were estimated according to the standards in the field.
Data exclusions	No data were excluded.
Replication	All attempts at replication were successful and the number of biological and technical replicates performed are mentioned in the text for each experiment.
Randomization	No experimental groups were involved hence randomization was not relevant to this study.
Blinding	No experimental groups were involved hence blinding was not relevant to this study.

Reporting for specific materials, systems and methods

We require information from authors about some types of materials, experimental systems and methods used in many studies. Here, indicate whether each material, system or method listed is relevant to your study. If you are not sure if a list item applies to your research, read the appropriate section before selecting a response.

Materials & experimental systems

n/a	Involved in the study	
☐	☒	Antibodies
☐	☒	Eukaryotic cell lines
☒	☐	Palaeontology and archaeology
☒	☐	Animals and other organisms
☒	☐	Human research participants
☒	☐	Clinical data
☒	☐	Dual use research of concern

Methods

n/a	Involved in the study	
☒	☐	ChIP-seq
☐	☒	Flow cytometry
☒	☐	MRI-based neuroimaging

Antibodies

Antibodies used	Primary antibodies:<br>- mouse monoclonal antibody α-GFP from Roche Diagnostics (#11814460001) used at a 1:1,000 dilution<br>- mouse monoclonal antibody α-PfGAPDH used at a 1:20,000 dilution<br>- rabbit α-PfHP1 used at a 1:5,000 dilution<br>Secondary antibodies:<br>- goat-α-mouse IgG (H&L)-HRP from GE Healthcare (#NXA931) used at a 1:10,000 dilution<br>- donkey anti-rabbit IgG (H&L) HRP GE Healthcare (#NA934) used at a 1:5,000 dilution
Validation	Mouse mAb α-PfGAPDH and rabbit α-PfHP1 Abs have been described in Refs. 90 and 91, respectively.<br>For the commercially available mAb α-GFP from Roche Diagnostics (#11814460001) and secondary goat α-mouse IgG (H&L)-HRP from GE Healthcare (#NXA931) and donkey anti-rabbit IgG (H&L) HRP GE Healthcare (#NA934) validation information is available from the manufacturers.

Eukaryotic cell lines

Policy information about cell lines

Cell line source(s)	The standard P. falciparum wild type strain NF54 (Delemarre and van der Kaay, Ned Tijdschr Geneeskd 1979; PMID: 390409) was obtained from Robert Sauerwein (Radboud University, Nijmegen, The Netherlands). The transgenic line NF54::DiCre (Tiburcio et al., mBio 2019; PMID: 31530668) was provided by Moritz Treeck (The Crick Institute, UK).
Authentication	None of the cell lines were authenticated in our lab.

April 2020

Mycoplasma contamination	The cell lines were not tested for mycoplasma contamination.
Commonly misidentified lines (See ICLAC register)	No commonly misidentified lines were used in this study.

Flow Cytometry

Plots

Confirm that:

- [x] The axis labels state the marker and fluorochrome used (e.g. CD4-FITC).
- [x] The axis scales are clearly visible. Include numbers along axes only for bottom left plot of group (a 'group' is an analysis of identical markers).
- [x] All plots are contour plots with outliers or pseudocolor plots.
- [x] A numerical value for number of cells or percentage (with statistics) is provided.

Methodology

Sample preparation	For parasite multiplication assays, synchronous NF54/AP2-G-mScarlet/CK2α-GFPDD parasites were split at 0-6 hpi (0.2 % parasitaemia) and cultured either in presence of 675 nM Shield-1 (+Shield-1) or in absence of Shield-1 (-Shield-1) during the whole duration of the multiplication assay. Synchronous NF54::DiCre/CK2α-GFP cKO parasites were split at 0-6 hpi (0.2% parasitaemia) and exposed for four hours to 100 nM rapamycin to trigger excision of the pfck2α-gfp gene (DMSO was added to the control population instead of rapamycin). Subsequently, the RBCs were washed once in PCM/2mM choline chloride and then resuspended in this medium for onward in vitro culture. After 18 hours (18-24 hpi; generation 1), 64 hours (18-24 hpi; generation 2), and 112 hours (18-24 hpi; generation 3) parasite DNA was stained at 37°C for 30 min using SYBR Green DNA stain (1:10,000, Invitrogen).<br>For schizogony and merozoite egress assays, synchronized NF54::DiCre/CK2α-GFP cKO parasites (0-4 hpi) were treated with RAPA or DMSO as explained above. Samples were collected for DNA content analysis starting at 20-24 hpi up to 20-24 hpi in the following generation. For merozoite egress experiments, 1 µM compound 2 (C2) was added to the cultures from 36-40 hpi onwards to prevent schizont rupture 54. At 50-54 hpi to 54-58 hpi, C2-arrested segmented schizont cultures were split and one half was directly inactivated by fixation in 4% formaldehyde/0.0075% glutaraldehyde (C2-arrested control). The other half of the sample was washed once in culture medium to remove C2, resuspended in culture medium and rotated at 37°C to allow merozoite egress and invasion for 45 min (replicate 1) and 45 min and 90 min (replicate 2) before samples were fixed in 4% formaldehyde/0.0075% glutaraldehyde. Fixed samples were washed twice in PBS and permeabilized for 15 min in PBS containing 0.1%Triton X-100 and 0.1 mg/ml RNase A. Fixed and permeabilized cells were washed twice in PBS and stained with SYBR Green DNA stain (1:5,000, Invitrogen) for 30 min.
Instrument	MACS Quant Analyzer 10 (Miltenyi Biotec)
Software	FlowJo_v10.6.1 software
Cell population abundance	200,000 cells were measured per sample. For each measurement we used uninfected red blood cells to be able to clearly determine the DNA-negative (i.e. uninfected) RBC population allowing us to gate and quantify the DNA-positive, parasite-infected RBC population.
Gating strategy	Gating was performed to remove small debris (smaller than cell size), doublets (single measurement event consisting of two cells) and to separate uninfected from infected RBCs (using an uninfected RBC control sample) based on their SYBR green intensity. Additional gate was set to separate mature schizonts from remaining iRBCs based on SYBR green intensity.

- [x] Tick this box to confirm that a figure exemplifying the gating strategy is provided in the Supplementary Information.
